# Predictors of knowledge and use of long-lasting insecticidal nets for the prevention of malaria among the pregnant women in Pakistan

**DOI:** 10.1186/s12936-021-03878-w

**Published:** 2021-08-23

**Authors:** Ramesh Kumar, Midhat Farzeen, Jamil Ahmed, Manohar Lal, Ratana Somrongthong

**Affiliations:** 1 Health Services Academy, Ministry of National Health Services Regulation and Coordination, Government of Pakistan, Islamabad, Pakistan; 2 Department of Family and Community Medicine, College of Medicine and Medical Sciences, Arabian Gulf University, Manama, Bahrain; 3 College of Public Health Sciences, Chulalongkorn University, Bangkok, Thailand

**Keywords:** Malaria awareness/knowledge, Pregnancy, Predicator, Long-lasting insecticidal nets, Mosquito bite prevention, Factors and healthcare workers, Pakistan

## Abstract

**Background:**

Malaria is endemic to Pakistan with high prevalence among pregnant women and linked with maternal anaemia, intrauterine growth retardation, preterm birth, and low birth weight. The use of long-lasting insecticidal nets (LLINs) is a proven and cost-effective intervention preventing malaria among pregnant women. The present study aimed to explore predictors of knowledge and use of LLINs among pregnant women in Pakistan.

**Methods:**

This was part of a quasi-experimental study of 200 pregnant women conducted in a rural district of Sindh province in Pakistan. Data were collected using Malaria Indicator Survey questionnaires developed by Roll Back Malaria Partnership to end Malaria Monitoring and Evaluation Reference Group. Pregnant women and mothers with newborns of six months of age were interviewed in their homes.

**Results:**

The age of the women was from 18 to 45, two thirds of the respondents (72.5%) were uneducated and married (77%). Majority (92%) of the women had received antenatal care during pregnancy, and 29.5% women had received counseling on malaria during their antenatal care visits. Multiple linear regression showed that the type of latrine was the most significant (β = 0.285, p < 0.001) determinant of knowledge about malaria among pregnant women followed by the death of a newborn (β = 0.271, p < 0.001). The use of mobile phone was the most significant (β = 0.247, p < 0.001) predictor of usage of LLINs among pregnant women followed by the death of a newborn (β = 0.232, p < 0.05).

**Conclusions:**

Maternal education, type of latrine, use of mobile phone, malaria during previous pregnancy and newborn death were strong predictors of knowledge and use of LLINs in pregnant women in Pakistan. There is a need to scale-up programmes that aim to create awareness regarding malaria among pregnant women. Mobile phone technology can be used to implement awareness programmes focusing on malaria prevention among women.

## Background

Malaria continues to be one of the major public health problems and sixth leading cause of death globally with 229 million cases annually, most in low-and-middle-income countries. Highest numbers of cases are reported in Africa followed by Eastern Mediterranean Region [1]. More than half of the population in the world is at substantial risk of malaria. An estimated 60% population currently lives in areas considered as moderately malaria endemic in Pakistan [1, 2], with about 3,500,000 suspected and confirmed cases identified annually. *Plasmodium falciparum* represents 15% of cases, and cumulative annual parasitic index (total number of positive slides for malaria in a year × 1000/Total population) in Pakistan is around 1.8 [3]. Multiple factors cause high transmission of malaria in Pakistan, including migration of people, low immunity, climatic changes variability, socio-economic conditions, weak health system, resource constraints, illiteracy, and suboptimal use of long-lasting insecticidal nets (LLINs) [3].

Pregnant women and children are at an increased risk of severe illness and deaths due to malaria [4]. Malaria is known to cause a high level of morbidity and mortality in both mothers and their newborn in low-and-middle-income countries [5]. Pregnant women may also have asymptomatic parasitaemia, and their newborns can also be exposed through placenta in malaria endemic zones [6]. Low birth weight, abortion, and miscarriage are serious health problems due to gestational anaemia resulting from malaria [7, 8]. These and other adverse outcomes during pregnancy can be worse in areas where *P. falciparum* or *Plasmodium vivax* are endemic [9, 10]. Pregnant women are, therefore, three times more at risk of dying due to malaria compared to non-pregnant women [11].

LLINs are an important preventive measure against malaria, however, they are used by one-fourth of the pregnant women in the world. Proper use of LLINs protects against malaria and transience from malaria [12–17]. Moreover, the frequency of malaria cases can be reduced to half among pregnant women only by using LLINs [14]. Use of LLINs may reduce reproduction number *R* (meant population where all individuals are susceptible to infection) of malarial parasites, which means if three quarters of the population uses LLINs, it could effectively eliminate malaria in the community [18]. Hence, proper use of LLINs in high endemic areas helps reduce malaria vector transmission and burden of malaria in the community [19]. LLINs act as a physical barrier and kill the mosquitoes offering a definitive shield against malaria [20]. Mass distribution of LLINs is a proven and cost-effective intervention to prevent malaria. Moreover, adequate knowledge and use of LLINs is considered an important factor against prevention of malaria [12, 21].

However, the challenges is that a mere availability of LLINs may not necessarily lead to their use, unless interventions focus upon knowledge that addresses the perceptions, misconceptions and promotes positive behaviours about the prevention of malaria [22]. The usefulness of LLIN dissemination in the community as an intervention is one of the strategies that can be complemented with its proper use by imparting knowledge, maintenance, and regular replacement. Hence, the World Health Organization (WHO) recommends assessing and evaluating the proper knowledge and use of LLINs to improve its use in the vulnerable communities facing a high burden of malaria. There is need to study factors capable of influencing knowledge and use of LLINs especially by vulnerable population groups like pregnant women in remote areas of Pakistan. This evaluation can provide useful insights about the gap between distribution of LLINs and their uptake by the communities. However, scarcely any previous studies have explored the factors that affect knowledge and use of LLINs among pregnant women in Pakistan. This study is aimed to determine the independent factors predicting the knowledge and use of LLINs to prevent malaria in pregnant women in rural Pakistan.

## Methods

This study was part of a larger interventional study employing quasi-experimental before and after design where baseline data were analysed before a health education intervention for malaria prevention among pregnant women of a rural district in the province of Sindh in Pakistan. The detailed methodology is published elsewhere [12]. Briefly, a sample of 200 pregnant women was recruited for data collection based on the sample size calculation with 80% power and alpha error of 0.50 to determine 30% difference. A multistage cluster random sampling technique was used to select study participants. First, union councils (smaller administrative areas in Pakistan) were selected from a list of 44 union councils in the district (primary sampling unit). Next, two union councils (Pathapur and Veeravah) were randomly selected for this study. In each union council, 10 villages were selected from a list of villages through simple random sampling method (secondary sampling unit), and in each village 10 pregnant women were selected through simple random sampling method from the list provided by the local Lady Health Workers. Pregnant women and mothers of children up to 6 months of age were part of the study who were interviewed at their homes. The women who were ill and did not belong to the study area at the time of the data collection were excluded. A validated and reliable Malaria Indicator Survey questionnaires developed by Roll Back Malaria Partnership Monitoring and Evaluation Reference Group was used in this study [3, 23].

Descriptive statistics were calculated for each knowledge and use of LLINs item. In order to test hypothesis that demographic/Independent variables (e.g., level of education, type of household, source of water) will predict knowledge and usage of LLINs among pregnant women, Multiple Linear Regression was used to predict knowledge about malaria and use of LLINS among pregnant women after controlling for age, level of education, mobile use, type of household, source of drinking water, type of latrine, mode of sewage, antenatal check-up, counselling about precaution against malaria during pregnancy, death of a newborn, malaria during previous and current pregnancy.

### Ethical approval

The study was approved by the Institutional Ethics Review Board of the Health Services Academy, Islamabad, Pakistan (F.No.7/82/2017-IERB). Written and informed consent was received from the participants before data collection.

## Results

The age of the women was from 18 to 45 years. Only (21%) women had completed ten years of school education and most (72.5%) were uneducated. The majority (77%) of the women were married at or earlier than 18 years of age. More than half (58.5%) of the women had from 3 to 4 children. Most (80.5%) women lived in mud houses consisting of three to four rooms, 62.5% did not own a mobile phone and 60% did not have an improved source of drinking water, 38% participants used open latrine, and 48.5% had open sewage drainage system in their households. Most (91.5%) of the women had monthly income from 5000 to 10,000 Pakistani rupees (USD = 33–65). Most (92%) of the women had history of making at least one antenatal care visit during their pregnancy and only 29.5% women received counseling on malaria during their antenatal care visit. Only a fifth of the women had history of malaria during their last pregnancy and only (1.5%) reported malaria during their current pregnancy (Table 1).

**Table. 1:** Baseline characteristics and information about malaria among pregnant women (n = 200)

Variables	Frequency	Percentage
Age		
< 20 years	17	8.5
20–25	44	22
26 and above	139	69.5
Education		
Uneducated	145	72.5
10 year	42	21
12 years and above	13	6.5
Number of living children		
1–2	27	13.5
3–4	117	58.5
5 and above	56	28
Death of a newborn		
Yes	44	22
No	156	78
Age at marriage		
18 and below	154	77
19 and above	46	23
Type of household		
Mud house	161	80.5
Brick house	39	19.5
Number of rooms		
1–2	51	25.5
3–4	149	74.5
Owns a mobile phone		
Yes	75	37.5
No	125	62.5
Source of drinking water		
A well outside home	120	60
A well inside home	49	24.5
Handpump	31	15.5
Type of latrine		
Open	76	38
Pit Latrine	123	61.5
Flush	1	0.5
Mode of sewage drainage in house		
Open sewers	97	48.5
Underground sewers	27	13.5
In open pond	49	24.5
No sewerage system	27	13.5
Income (PKR)		
5000–10,000	183	91.5
11,000–20,000	17	8.5
Antenatal checkup		
Yes	184	92
No	16	8
No. of antenatal visits		
1	15	7.5
2	95	47.5
3	38	19
4	36	18
Did not answer	16	8
ANC counseling on malaria		
Yes	59	29.5
No	141	70.5
Malaria during any previous pregnancy		
Yes	42	21
No	158	79
Malaria during the current pregnancy		
Yes	3	1.5
No	197	98.5

Multiple linear regression was used to predict knowledge about malaria among pregnant women based on the independent variables (e.g., age, level of education). A significant regression equation was found [F (12, 187) = 5.779; p < 0.001] with an R^2^ i.e., amount of variance explained by independent variables was 0.271 or 27%. However, it can be seen that out of 12 independent variables (IVs), only five variables added statistically significant difference to the prediction of knowledge, i.e., level of education, us of mobile phone, type of latrine, malaria during previous pregnancy and newborn death. Furthermore, it can be inferred that the type of latrine was the most significant (β = 0.285, p < 0.001) determinant of knowledge about malaria among pregnant women followed by the death of a newborn (β = 0.271, p < 0.001) as shown in (Table 2).

**Table. 2:** Multiple linear regression analysis to predict Knowledge of pregnant women regarding malaria

Variables	R	R^2^	∆ R^2^	Β	SE	β	t	Sig(p)
	0.520	0.271	0.224					0.014*
Age				0.159	0.234	0.049	0.680	0.497
Level of education				− 0.647	0.177	− 0.262	− 3.648	0.000**
Mobile phone use				0.973	0.403	0.166	2.416	0.017*
Type of household				− 1.037	0.719	− 0.151	− 1.442	0.151
Source of drinking water				− 0.066	0.434	− 0.017	− 0.152	0.879
Type of latrine				1.635	0.487	0.285	3.357	0.001**
Mode of sewage				0.126	0.246	0.050	0.512	0.609
Antenatal check-up				− 0.797	0.747	− 0.076	− 1.067	0.287
Counselling about precaution against malaria during pregnancy				0.071	0.595	0.011	0.119	0.906
Death of new born				1.854	0.575	0.271	3.225	0.001**
Malaria during previous pregnancy				− 1.389	0.497	− 0.208	− 2.793	0.006**
Malaria during current pregnancy				1.476	1.549	0.063	0.953	0.342

*R*^2^ amount of variance explained by IVs,* ∆R*^2^ additional variance in DV,* B* unstandardized coefficient,* SE*Standard Error,* β* Standardized coefficient, *t* estimated coefficient

*p < 0.05, ** p < 0.01

Similarly, multiple linear regression was also calculated to explore the most significant independent variables that can predict usage of LLINs among pregnant women. As shown in Table 3, a significant regression equation was found [F (12, 187) = 4.579; p < 0.001] with an R^2^ i.e. amount of variance explained by independent variables is 0.178 or 19%. Additionally, as with Knowledge, same independent variables had variance, which was significant (p < 0.05) to predict usage of LLINs. Furthermore, use of mobile phone was most significant (β = 0.247, p < 0.001) predictor of usage of LLINs among pregnant women followed by death of a newborn (β = 0.232, p < 0.05).

**Table. 3:** Multiple Linear Regression analysis to analysis to predict Use of LLINs among pregnant women

Variables	R	R^2^	∆ R^2^	Β	SE	β	t	Sig(p)
	0.477	0.227	0.178					0.001**
Age				0.082	0.184	0.033	0.445	0.657
Level of education				− 0.381	0.138	− 0.202	− 2.735	0.007**
Mobile phone use				1.102	0.316	0.247	3.488	0.001**
Type of household				− 0.739	0.564	− 0.141	− 1.311	0.191
Source of drinking water				0.183	0.340	0.063	0.537	0.592
Type of latrine				0.904	0.382	0.207	2.367	0.019*
Mode of sewage				− 0.088	0.193	− 0.046	− 0.454	0.651
Antenatal check-up				− 0.630	0.586	− 0.079	− 1.076	0.283
Counselling about precaution against malaria during pregnancy				0.168	0.467	0.036	0.361	0.719
Death of a newborn				1.210	0.451	0.232	2.683	0.008**
Malaria during previous pregnancy				− 0.983	0.390	− 0.193	− 2.521	0.013*
Malaria during current pregnancy				− 0.345	1.215	− 0.019	− 0.284	0.777

*p < 0.05, ** p < 0.01

*R*^2^ amount of variance explained by IVs, *∆R*^2^ additional variance in DV, *B* unstandardized coefficient, *SE* Standard Error, *β* Standardized coefficient, *t* estimated coefficient

## Discussion

Maternal education, type of latrine, use of mobile phone, malaria during previous pregnancy and newborn death were significant predictors of knowledge and use of LLINs among pregnant women in this study. This study was conducted in the rural area of Tharparkar, a high endemic area with significant malaria infection, high poverty, illiteracy and poor overall infrastructure development status. Studies have also supported the findings and shows a clear link between maternal education and poverty, and incidence of malaria in pregnant women and their newborns [22, 24]. The use of LLINs has been shown to be higher in educated mothers and those receiving regular antenatal care and counselling [12, 25]. A study from Nigeria showed that maternal education can effectively prevent malaria in rural communities [26]. Female education is crucial to the prevention of malaria in pregnancy [27]. Successful Health education campaigns can encourage the use of LLINs [28]. Educational interventions can also improve knowledge about malaria leading to a higher use of LLINs [15]. Evidence shows that educational interventions can improve the use of LLINs by 30% in rural communities [29].

Type of latrine used by the community was another strong factor determining both knowledge and use of LLINS among pregnant women in this study. However, most of the study population was using the pit latrine with open drainage system at the time of this study. This type of latrine use can lead to problems like leakage of water from wastewater drain, which can create suitable environment and breeding ground for the growth of mosquitoes leading to a high incidence of malaria in these communities. The findings of this study are supported by the existing evidence showing that poor sanitation is directly linked with high incidence of parasitic infections and vector borne diseases including malaria [30, 31]. Improved toilet use, like the use of flush toilet with piped water, is linked with a reduction in the incidence of malaria [32].

Mobile phone use among the pregnant women is found positive predictor for knowledge and use of LLINs. This finding is supported by an experimental study proved that the mobile phone short messages are an effective intervention to prevent malaria [33]. Mobile health interventions are strongly associated on health outcomes if the messages are constructed to affect people’s behavior. Moreover, malaria treatment adherence and awareness may be increased through regular mobile phone messages in malaria patients. The findings of present study are consistent with these studies [34].

Malaria during previous pregnancy and newborn death were strong predictors of the knowledge and use of LLINs among pregnant women in this study. This could be because of the knowledge gained about the transmission of malaria during previous pregnancy resulting in the death of a newborn. This finding is supported by a longitudinal survey that showed that the newborns are at high risk of death and observed a significant reduction in mean birth weight in babies of women with malaria during pregnancy. Malaria during pregnancy was a predictor of knowledge and use of LLINs, and it is a known risk of adverse pregnancy outcomes like miscarriage, preterm birth, stillbirth, and low birth weight leading to high infant mortality [5, 35]. The finding of previous malaria infection strongly underscores the need for preventive efforts during malaria. The newborn mortality and premature birth are a known consequence of malaria in pregnancy [36]. Conditions like low birth weight in turn cause intrauterine growth retardation and preterm birth, which are leading causes of newborn mortality in low- and middle-income countries [7, 37]. These findings are reported from the baseline data analysed before the health education intervention for malaria prevention among pregnant women of the same rural area published elsewhere [12]. Based on the trends shown in this baseline study, comparable results are expected if the intervention is implemented at a larger scale. Since the respondents were selected from separate UCs, it could be assumed that due to a sufficient distance between the two areas, any possibilities of contamination were minimum. Sample selection by simple random sampling method was a strength of this study. Another strength of this study was that the use of multiple linear regression analysis has predicted the factors, which are validated by the existing knowledge and use of LLINs for the prevention of malaria.

This study cannot be generalized to the whole population in Pakistan the sample was selected from only one district. Moreover, limited funds and time constraints were the major limitations of this study. Further evidence may be generated through community trials to test the effectiveness of these factors/ predictors and also understand the host-seeking behaviour and response of mosquitoes to LLINs.

## Conclusions

The education level, mobile phone usage, type of latrine, death of a newborn and malaria during previous pregnancy were most significant predictors of knowledge and use of LLINs among pregnant women of rural Pakistan. Therefore, there is a need to scale-up programmes, which aim at creating awareness regarding malaria among pregnant women. This analysis may help streamline interventions to enhance uptake of LLINs (along with intermittent preventive treatment in pregnancy) [38]. It is recommended that health policy makers may focus on female education and start awareness programmes on malaria prevention through regular mobile phone messages. Mobile phone technology could be used as a strategy for controlling malaria in high-risk group, i.e., pregnant women.

## Data Availability

The datasets used and/or analysed during the current study are available from the corresponding author on reasonable request.

## Abbreviations

EMRO: Eastern Mediterranean regional Office
UC: Union council
LLINs: Long-lasting insecticidal nets
LHW: Lady Health Worker
BHU: Basic health unit
TDR: Tropical disease research
WHO: World Health Organization
